# Multifaced roles of cannabinoid therapy in cancer: balancing analgesia, antitumor potential, and systemic toxicity

**DOI:** 10.3389/fphar.2025.1691893

**Published:** 2025-11-13

**Authors:** Ioana Creanga-Murariu, Mitica Ciorpac, Raluca-Maria Gogu, Cosmin-Vasilica Pricope, Veronica Bild, Daniela-Carmen Ababei, Leontina-Elena Filipiuc, Andrei Szilagyi, Claudiu-Laurentiu Josan, Irina-Draga Caruntu, Ludmila Lozneanu, Andrei Timofte, Carmen Solcan, Dragos-Viorel Scripcariu, Peter Hegyi, Teodora Alexa-Stratulat, Bogdan-Ionel Tamba

**Affiliations:** 1 Advanced Research and Development Center for Experimental Medicine (CEMEX), Grigore T. Popa University of Medicine and Pharmacy, Iasi, Romania; 2 Medical Oncology-Radiotherapy Department, Grigore T. Popa University of Medicine and Pharmacy, Iasi, Romania; 3 Centre for Translational Medicine, Semmelweis University, Budapest, Hungary; 4 Department of Biology, Faculty of Biology, Alexandru Ioan Cuza University of Iasi, Iasi, Romania; 5 Radiology Department, “Sf. Spiridon” Hospital, Iasi, Romania; 6 Pharmacodynamics and Clinical Pharmacy Department, Grigore T. Popa University of Medicine and Pharmacy, Iasi, Romania; 7 Department of Morpho‐Functional Sciences I, Grigore T. Popa University of Medicine and Pharmacy, Iasi, Romania; 8 Romanian Medical Science Academy, Bucharest, Romania; 9 Faculty of Veterinary Medicine, “Ion Ionescu de La Brad” University of Life Sciences, Iasi, Romania; 10 Department of Surgery, Grigore T. Popa University of Medicine and Pharmacy, Iasi, Romania; 11 Institute for Translational Medicine, Medical School, University of Pécs, Pécs, Hungary; 12 Institute of Pancreatic Diseases, Semmelweis University, Budapest, Hungary; 13 Translational Pancreatology Research Group, Interdisciplinary Centre of Excellence for Research Development and Innovation, University of Szeged, Szeged, Hungary

**Keywords:** cannabinoid, cancer, pain, breast cancer, antitumor, cachexia

## Abstract

**Introduction:**

Cannabinoids hold promise in oncology for symptom relief and antitumor effects, though concerns about safety and efficacy persist. This study assessed the impact of JWH-182 and phytocannabinoids NC1 – Cannabixir® Medium dried flowers and NC2 – Cannabixir® THC full extract, in a murine breast cancer model with paclitaxel-induced peripheral neuropathy (CIPN).

**Methods:**

Female BALB/c mice with breast tumors received paclitaxel alone or combined with cannabinoids, and outcomes included pain sensitivity, tumor progression (imaging and histopathology), cachexia (body weight, food intake, imaging), as well as hematological and organ toxicity profiles.

**Results:**

All cannabinoids alleviated neuropathic pain, with NC1 most effective for central and thermal protection (72% and 100%, p < 0.0001), NC2 showing strong central and mechanical benefit (>60% and >33%), and JWH-182 intermediate (∼50%). Tumor growth was not significantly altered, but metastasis incidence was 41.7% for NC1, 58.3% for NC2, compared with 70% for PTX, suggesting antitumoral activity. Effects on cachexia were modest, JWH-182 tended to improve food intake, whereas NC1 and NC2 reduced it, yet body weight remained stable and significant muscle loss was observed only with NC2 (p < 0.05). Hematology showed immunomodulatory effects, with cannabinoids reversing lymphopenia (p = 0.0005), raising monocytes and neutrophils, and partly restoring platelets. Toxicity was highest with NC2 (renal and hepatic injury), moderate with NC1, and lowest for kidney with JWH-182 but with greater hepatic inflammation.

**Conclusion:**

Cannabinoids show potential in oncology by relieving CIPN and influencing tumor dynamics, with mostly neutral effects on cachexia. GMP-certified formulations enhance translational value, though safety concerns warrant further study.

## 1 Introduction

By 2040, cancer is projected to become the most common global disease, with over 29 million cases, largely due to aging and increased risk exposure (Siegel et al., 2023). In the U.S., cancer-related healthcare costs are expected to reach $246 billion by 2030, with 52.2% of FDA-approved drugs between 2000 and 2017 targeting cancer (Batta et al., 2020).

Despite therapeutic advances, chemotherapy remains limited by severe side effects and resistance. Chemotherapy-induced peripheral neuropathy (CIPN), caused by nerve damage, often leads to dose reduction or treatment discontinuation (Lyman, 2009). Current CIPN treatments focus on symptom relief but have inconsistent efficacy (Loprinzi et al., 2020). Resistance and toxicity further complicate chemotherapy, even as it remains central to most first-line regimens.

Cannabinoids have emerged as promising agents in oncology for both symptom relief and potential antitumor effects (Creanga-Murariu et al., 2023). By acting on cannabinoid receptors 1 and 2 (CB1R, CB2R), Tetrahydrocannabinol (THC) and Cannabidiol (CBD) help regulate pain, appetite, and inflammation, making them effective in managing CIPN, cancer pain, and cachexia (Boggs et al., 2016). Preclinical studies also suggest that cannabinoids can inhibit tumor growth, metastasis, angiogenesis, and reverse chemoresistance, with potential to enhance chemotherapy efficacy and reduce its toxicity (Velasco et al., 2016a). Although generally well tolerated, the long-term safety, toxicity, and drug interactions of cannabinoids remain underexplored (Velasco et al., 2016b). With rising patient interest and encouraging evidence, cannabinoids represent a promising adjunct in cancer care, warranting further investigation.

This study uses a murine model of breast cancer with paclitaxel-induced peripheral neuropathy to evaluate the therapeutic potential of one synthetic cannabinoid (JWH-182) and two phytocannabinoid formulations (Cannabixir® Medium dried flowers, 15.6% THC: <1% CBD (NC1), and Cannabixir® THC full extract, (NC2)) in relieving neuropathic pain and exploring synergistic effects with paclitaxel on tumor progression. Secondary objectives include evaluating their role as adjuvant therapies for cancer-associated cachexia, as well as their impact on quality of life, survival, and toxicity.

## 2 Materials and methods

### 2.1 Drugs formulation and administration

JWH-182 (Cayman Chemical) was dissolved in saline:Polysorbate 80 (99:1, v/v) and given intraperitoneally (0.20 mL/10 g). Paclitaxel (Sigma Aldrich, Germany) was prepared in saline:Cremophor:ethanol (99:0.5:0.5, v/v/v) and administered intraperitoneally (0.10 mL/10 g). NC1 (Cannabixir®, Cansativa GmbH, Germany) dried flowers, 15.6% THC: <1% CBD, were ground, sieved (125 µm), suspended in sodium carboxymethyl cellulose:water (0.1:99.9, m/v), and given orally (0.20 mL/10 g). NC2 (Cannabixir ®, FYTA Company B.V., Netherlands), full THC extract, was suspended in water:Polysorbate 80 (99:1, v/v) and administered orally (0.20    mL/10 g).

### 2.2 Cells and media

The 4T1 cell line (triple negative breast cancer) was obtained from ATCC (Virginia, United States). DMEM, FBS, Penicillin/Streptomycin, Trypsin-EDTA, MTT powder, and DMSO were purchased from Sigma-Aldrich (Saint Louis, United States).

### 2.3 Animals and housing conditions

Female BALB/c mice (8–10 weeks, 15–25 g) were purchased from National Institute for Medical-Military Research and Development “Cantacuzino” (Bucharest, Romani) and housed in the CEMEX facility (“Grigore T. Popa” Medicine and Pharmacy University, Iaşi) under controlled conditions (20 °C ± 4 °C, 50% ± 5% humidity, 12 h light-dark cycle) in enriched, individually ventilated cages, with *ad libitum* access to food and water. Isoflurane (ISOFLUTEK 1000 mg/g inhalation vapour, liquid) used for animals’ anaesthesia was obtained from Laboratorios KARIZOO, S.A. (Caldes de Montbui, Spain) through the holder of the marketing authorization in Romania, Maravet SA (Baia Mare, Romania). At the end of experiment, anesthetised animals were euthanized by rapid decapitation. The experimental study was carried out in compliance with the ARRIVE guidelines, European Directive 2010/63/EU and AVMA Guidelines for the Euthanasia of Animals (2020) (Percie du Sert et al., 2020) and it was authorized by the university’s Research Ethics Committee (no. no. 342/7.09.2023) and the Romanian National Sanitary Veterinary and Food Safety Authority (no. 70/26.01.2024).

### 2.4 Experimental design

#### 2.4.1 *In vitro* toxicity test

Three thousand 4T1 breast cancer cells (stage IV) were seeded per well in 96-well plates and incubated for 24 h at 37 °C, 5% CO_2_. Cells were then treated for 24 h with increasing concentrations (5–25 µM) of each cannabinoid or paclitaxel (0.1–10 µM). Controls received 0.1% DMSO to match solvent exposure. Cell viability was assessed using the MTT assay in triplicate, with absorbance measured at 570 nm (EZ Read 400, Biochrom, United Kingdom). Viability was expressed as a percentage of untreated controls, and IC_50_ values were calculated. In a second phase, cells were treated with cannabinoid-paclitaxel combinations based on their respective IC_50_ concentration.

#### 2.4.2 *In vivo* study overview

**FIGURE 1:**
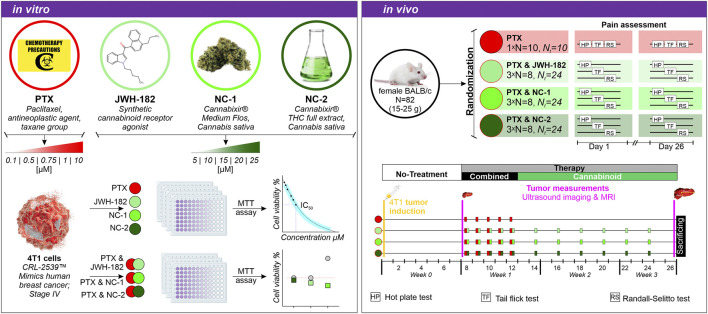
Experimental design. *In vitro:* 4T1 breast cancer cells were treated with PTX, JWH-182, NC-1, and NC-2, alone or in combination and cell viability was assessed via MTT assay. *In vivo:* BALB/c mice were randomized into four treatment groups (PTX alone or combined with cannabinoids), and pain was assessed using the Hot Plate, Tail Flick, and Randall-Selitto tests on days 1, 8, and 26. Tumor progression was monitored at baseline and end of experiment via breast ultrasound and MRI scans.

As shown in Figure 1, healthy mice were randomized and baseline pain assessed using Hot Plate, Tail Flick, and Randall-Selitto tests. All groups received 4T1 xenografts on day 1. On day 8, after tumors became palpable, baseline tumor volume was measured via ultrasound and MRI. Treatments began with a single cannabinoid dose (day 8), followed by 4 days of co-administration with paclitaxel (days 9–12), then cannabinoids every other day for 14 days. On day 26, pain tests and tumor measurements were repeated, and animals were sacrificed for blood and tissue collection.

#### 2.4.3 Randomization, tumor induction and treatment administration

Healthy female BALB/c mice were allocated to four treatment groups using block randomization to ensure balanced distribution throughout the study: 24 animals for the JWH-182; 24 for NC1; 24 for NC2 and finally 10 animals for the PTX (control). Then, animals in the cannabinoid groups were further randomized in three equal lots (8 individuals), corresponding to one type of pain test. Consequently, eight animals in the JWH-182, NC1 and NC2 respectively were subjected to Hot Plate, the same number of animals were randomized to Tail Flick and finally to Randal Selitto pain test. The animals in the PTX group were subjected to all the pain tests in three different days. Following baseline pain tests, orthotopic 4T1 breast tumors were induced. 4T1 cells were cultured in DMEM with FBS and Penicillin/Streptomycin, harvested at 80%–90% confluency, and resuspended at 1 × 10^5^ cells/50 µL. Under isoflurane anesthesia, 50 µL was injected into the 6th left mammary fat pad. A palpable tumor developed within 7 days. All animals received 2 mg/kg paclitaxel intraperitoneally, a dose known to induce peripheral neuropathy, as previously validated by our group (Polomano et al., 2001; Flatters and Bennett, 2006). Neuropathy onset was observed by day 10, peaking at day 18 (Filipiuc et al., 2024a). Cannabinoid doses were based on effective dose (ED) values from our prior CIPN studies (Filipiuc et al., 2024a). NC1 (75–600 mg/kg) (Filipiuc et al., 2024b) and NC2 (8.1–129.5 mg/kg) were given orally, with EDs of 75 mg/kg and 12.72 mg/kg, respectively. JWH-182 was given intraperitoneally (0.63–5 mg/kg), with an ED of 2.63 mg/kg (Filipiuc et al., 2024a). Controls received paclitaxel and saline only. Treatment included one cannabinoid induction dose post-tumor detection, 4 days of co-administration with paclitaxel, then cannabinoids every other day for 14 days.

### 2.5 Thermal and mechanical induced pain sensitivity assays

To establish baseline pain thresholds, healthy animals underwent three pain tests, performed by a blinded assessor: Hot Plate, Tail Flick, and Randall-Selitto. In the Hot Plate test, mice were placed on a 52.5 °C ± 0.1 °C heated surface, and response latency was recorded (30 s cut-off). In the Tail Flick test, radiant heat (52 °C ± 0.1 °C) was applied to the tail, with a 15 s cut-off. For both, analgesic effect was expressed as % Maximum Possible Effect (% MPE): % Inhibition = [(Tx–T0)/(Tm–T0)] × 100, where T0 = baseline latency, Tx = post-treatment latency, and Tm = cut-off. In the Randall-Selitto test, increasing pressure was applied to the paw, with pain response calculated as % MPE using the formula: % Inhibition = [(gx–g0)/(gm–g0)] × 100, where g0 = baseline latency, gx = post-treatment latency, and gm = cut-off weight. At the end of the treatment period, animals were retested using the same procedures. Based on % MPE, animals were grouped into pain perception categories: allodynia (−100% to −30%), normal pain sensitivity (−30 to +30%), and hypoalgesia (+30 to +100%). These thresholds, grounded in standardized pain research practices, allowed objective classification of pain responses and visualization of the treatment’s impact on pain sensitivity. After calculating % Inhibition for each pain test (Hot Plate, Tail Flick, and Randall-Selitto), animals were categorized into three pain sensitivity groups based on their deviation from baseline thresholds. Values between −30% and +30% indicated normal pain perception, reflecting minimal change in sensitivity. Values from −100% to −30% were classified as allodynia, suggesting increased sensitivity to thermal or mechanical stimuli. Values between +30% and +100% indicated hypoalgesia, characterized by reduced pain sensitivity. These thresholds were chosen to provide a standardized, objective classification of pain responses, consistent with established pain research methodologies (Fillingim et al., 2016). The resulting data allowed visualization of the density distribution of pain responses across groups, offering a comprehensive view of treatment effects and the extent to which pain thresholds were restored to baseline levels.

### 2.6 Tumor volume & specific growth rate assessment

Tumor growth was blindly assessed at baseline and post-treatment using breast ultrasound and MRI. Ultrasound imaging was performed with the VisualSonics Vevo® 2,100 system and a 30 MHz probe, under isoflurane anesthesia (3% induction, 2% maintenance). Mice were positioned supine, the tumor area was shaved, and three-dimensional measurements (anteroposterior, transverse, craniocaudal) were recorded and archived for analysis. Whole-body MRI was conducted using a 1T nanoScan® PET/MRI scanner with a 35-mm coil. Two sequences were acquired: axial T1 GRE (TR = 12.1 ms, TE = 3.3 ms, FA = 15°, 80 slices, 1 mm thickness, FOV = 35 × 36 mm, resolution = 0.109 × 0.25 mm^2^) and axial T2 FSE (TR = 17,286 ms, TE = 48.6 ms, ETL = 16, FOV = 34 × 35 mm, resolution = 0.227 × 0.273 mm^2^). Imaging lasted ∼15 min per animal. Images were analyzed with InterView Fusion software, recording tumor size and morphology, and assessing lung and liver metastases.

The tumor-specific growth rate (SGR) was determined under the assumption of exponential tumor growth, where the rate of increase is proportional to tumor volume (Mehrara et al., 2007). SGR, expressed as the percentage increase in volume per day, was calculated using the formula described by Mehrara et al. (2007): *SGR [%/day] =* ln*(V*_2_ */V*_1_ *)/(t*_2_ *- t*_1_ *),* where *V*_1_ is the tumor volume at the first time point (*t*_1_) and *V*_2_ is the tumor volume at the end of the observation period (*t*_2_). For the pre-treatment period (day 1–8), we assumed an arbitrary initial tumor volume (*V*_1_) of 1 mm^3^ at the time of tumor model induction. The tumor volume (*V*_2_) was determined using two imaging methods—ultrasound (US) and magnetic resonance imaging (MRI)—at day 7 after tumor induction. For the treatment period (day 8–26), the tumor SGR was calculated using the tumor volumes determined by both imaging methods at day 7 (*t*_1_) and at the end of the observation period on day 26 (*t*_2_).

### 2.7 Neoplastic cachexia assessment

Cancer-associated cachexia was assessed by tracking weekly body weight, food intake, and muscle mass via MRI. Body weight was measured weekly from baseline to study end. Food intake was recorded every other day using pre-weighed pellets (20 g); leftover and spilled food was weighed after 48 h to calculate consumption. Muscle loss was evaluated through MRI analysis of psoas and paraspinal muscles (Morrell et al., 2016) in all study animals. A single axial slice at the L4–L5 level was used to measure cross-sectional muscle area. Manual regions of interest (ROIs) were drawn bilaterally using consistent anatomical landmarks, and segmentation was performed using InterView Fusion software (Mediso nanoScan® v.20).

### 2.8 Hematology, necropsy and histopathology (toxicity and metastatic rate assessment)

On day 26 post-tumor induction, all animals were euthanized and blood was collected via decapitation in 3 mL vacutainer tubes for hematology. A full necropsy was performed, examining the external body, orifices, and internal cavities. The organosomatic index (OSI) was calculated as the ratio of organ weight (tumor, brain, lung, liver, kidney, spleen) to total body weight, expressed as a percentage. To assess cumulative hematological toxicity from cannabinoids and paclitaxel, blood samples were analyzed within 30 min using the HEMAVET 950 analyzer (Drew Scientific). Complete blood counts included leukocytes, neutrophils, eosinophils, basophils, monocytes, lymphocytes, erythrocytes, hemoglobin, hematocrit, MCV, MCH, MCHC, reticulocytes, and platelets. Liver and kidney tissues were fixed in 10% formalin for detailed histopathological analysis. Organs were trimmed according to standardized mouse sampling guidelines (Ruehl-Fehlert et al., 2003), then processed using the Excelsior™ AS Tissue Processor and embedded in paraffin blocks. Each block was sectioned at 4 µm using a semi-automatic microtome (CUT 5062), and two sections per organ were stained with hematoxylin and eosin (H&E). Slides were scanned at ×400 magnification using an Aperio AT2 DX slide scanner, and photomicrographs were analyzed for histological changes indicative of drug-induced toxicity, compared to control samples. To assess metastatic spread, digital histology images of the liver and lungs were evaluated for tumor cell invasion. Metastatic foci were identified based on characteristic morphology and manually counted per tissue section. Metastasis presence was recorded as a binary outcome (present/absent), and the number of lesions per animal was noted. The metastasis rate was calculated as the percentage of animals with detectable metastases in each organ.

### 2.9 Statistical evaluation

Statistical analyses were performed using R software and relevant packages tailored to each dataset and hypothesis. Normality of data distribution was assessed using the Shapiro-Wilk test, and homogeneity of variances was evaluated as needed. For comparisons among groups, parametric tests such as ANOVA or non-parametric tests, including the Kruskal-Wallis test and Wilcoxon rank-sum test, were applied depending on the data’s distribution. Post hoc analyses included pairwise t-tests or pairwise Wilcoxon tests with Bonferroni correction for multiple comparisons. Chi-square tests and Z-test for proportions were utilized for categorical data analysis. Visualization of results was implemented using the ggplot2, ggpubr, and related R libraries, ensuring clarity of data representation. Detailed analyses and scripts for each figure are available upon request.

## 3 Results

### 3.1 The effect of cannabinoids on chemotherapy-induced neuropathy

The neuroprotective effect of cannabinoids was evaluated using three types of pain-inducing tests, each analyzing a different mechanism of pain processing. We found that the response to pain stimulus was not homogeneous among individuals, however it followed some patterns.

**FIGURE 2:**
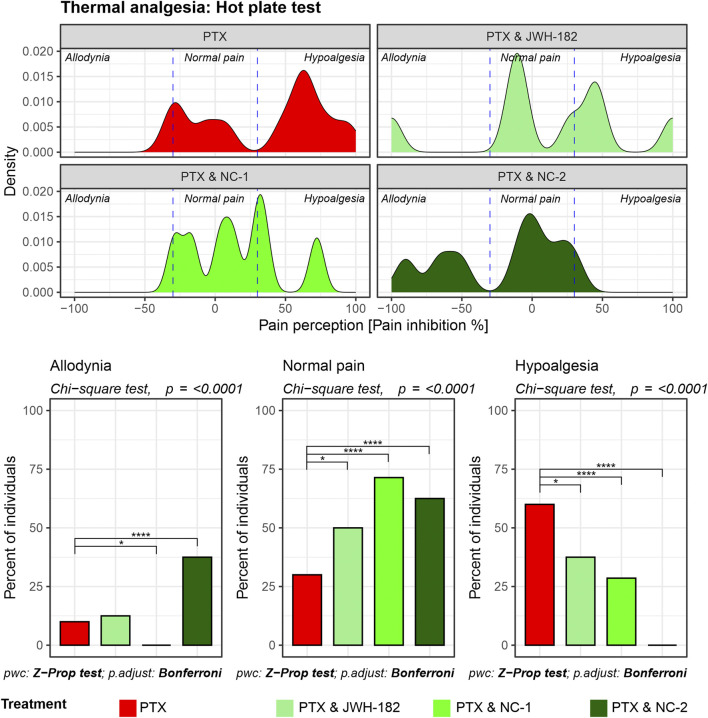
Thermal nociception as assessed by the Hot Plate test. Density plots display the distribution of pain perception across three categories: allodynia, normal pain, and hypoalgesia. PTX alone results in significant hypoalgesia, while the addition of cannabinoids maintains baseline pain thresholds, reducing the risk for both hypoalgesia and allodynia. Bar graphs highlight statistically significant differences between treatment groups in all three pain categories (Chi-square test, p < 0.0001).

In the case of animals undergoing central pain stimulation with the use of the hot plate test (Figure 2), two-thirds of the animals treated with PTX showed hypoalgesia after treatment, confirming the presence of neuropathy and nerve damage induced by chemotherapy. By contrast, animals treated with both PTX and NC1 reacted differently, with 72% of the individuals exhibiting a normal response to pain stimulus, similar to their own baseline values, suggesting neuroprotection (p < 0.0001), while only 28% showed signs of neuropathy (p < 0.0001). Moreover, more than 60% of animals treated with NC2 showed the same feature of maintaining the baseline pain threshold (p < 0.0001), while the rest presented with allodynia, translated as irritated, but not completely damaged nerve fibers (p < 0.0001). Similar results were noted in animals treated with the synthetic cannabinoid JWH-182 which showed different patterns of pain responses, with half of them presenting normal pain threshold.

**FIGURE 3:**
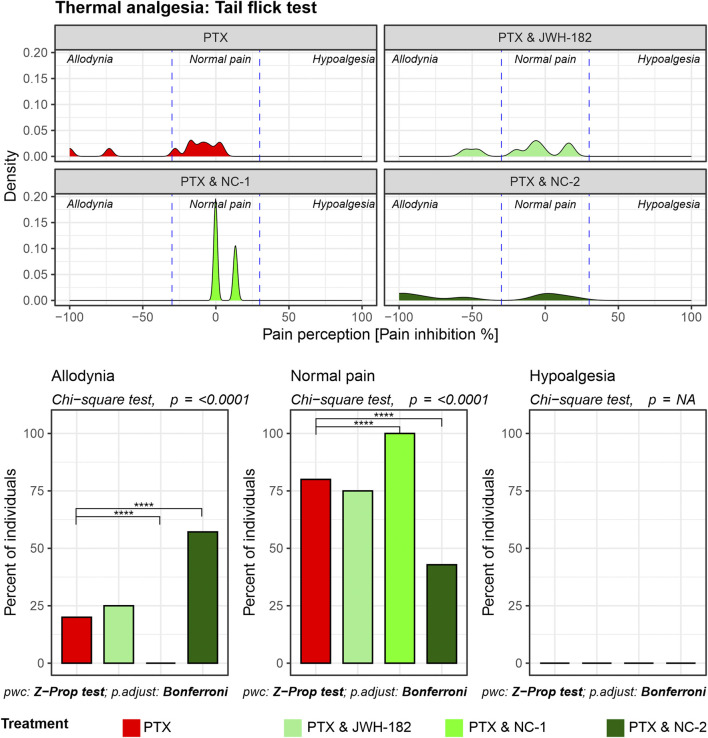
Spinally mediated nociception assessed using the Tail Flick test. Density plots depict pain perception distributions across allodynia, normal pain, and hypoalgesia categories, demonstrating that PTX alone causes pronounced allodynia with minimal restoration of normal pain thresholds. Cannabinoid combinations significantly reduce allodynia and restore normal pain thresholds, as highlighted in the bar graphs (Chi-square test, p < 0.0001).

The tail flick test results (Figure 3) revealed that reflex nociceptive pathway is less affected by PTX, with only 25% of individuals experiencing allodynia after treatment exposure. Even so, NC1 showed the same ability to protect the nerve fibers, with all the animals presenting with the same pain response behavior, compared to their own baseline thresholds and with PTX-only treated animals (p < 0.0001). The same shifting towards normalized pain response applies after treatment with NC2 or JWH-182, however the number of animals is smaller.

**FIGURE 4:**
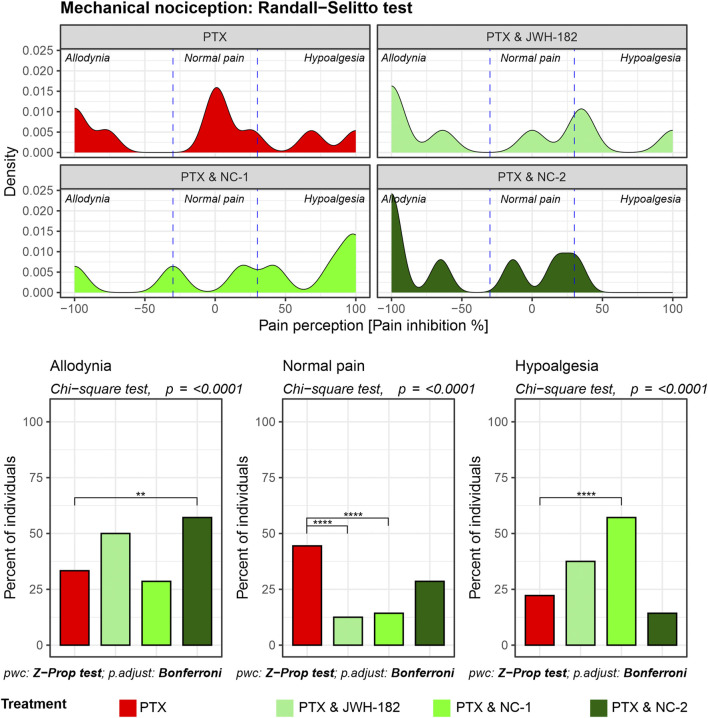
Mechanical nociception assessed using the Randall-Selitto test. Density plots depict the distribution of pain perception across allodynia, normal pain, and hypoalgesia, showing that PTX treatment results in significant allodynia and hypoalgesia NC-2 demonstrates superior efficacy in alleviating mechanical hypersensitivity compared to other cannabinoids, highlighting its strong peripheral anti-inflammatory and neuroprotective effects.

The Randall-Selitto test showed that more than half of the PXT-treated animals had abnormal pain perception, either allodynia or hypoalgesia, at the end of the experiment. Similarly, both JWH-182 and NC1 combinations led to changes in mechanical pain perception. NC2 showed the best results among the cannabinoids, with more than one-third of animals shifting out of the allodynia or hypoalgesia zones, getting back their normal pain responses (Figure 4).

### 3.2 Antitumor activity of cannabinoids

#### 3.2.1 *In vitro* assessment

**FIGURE 5:**
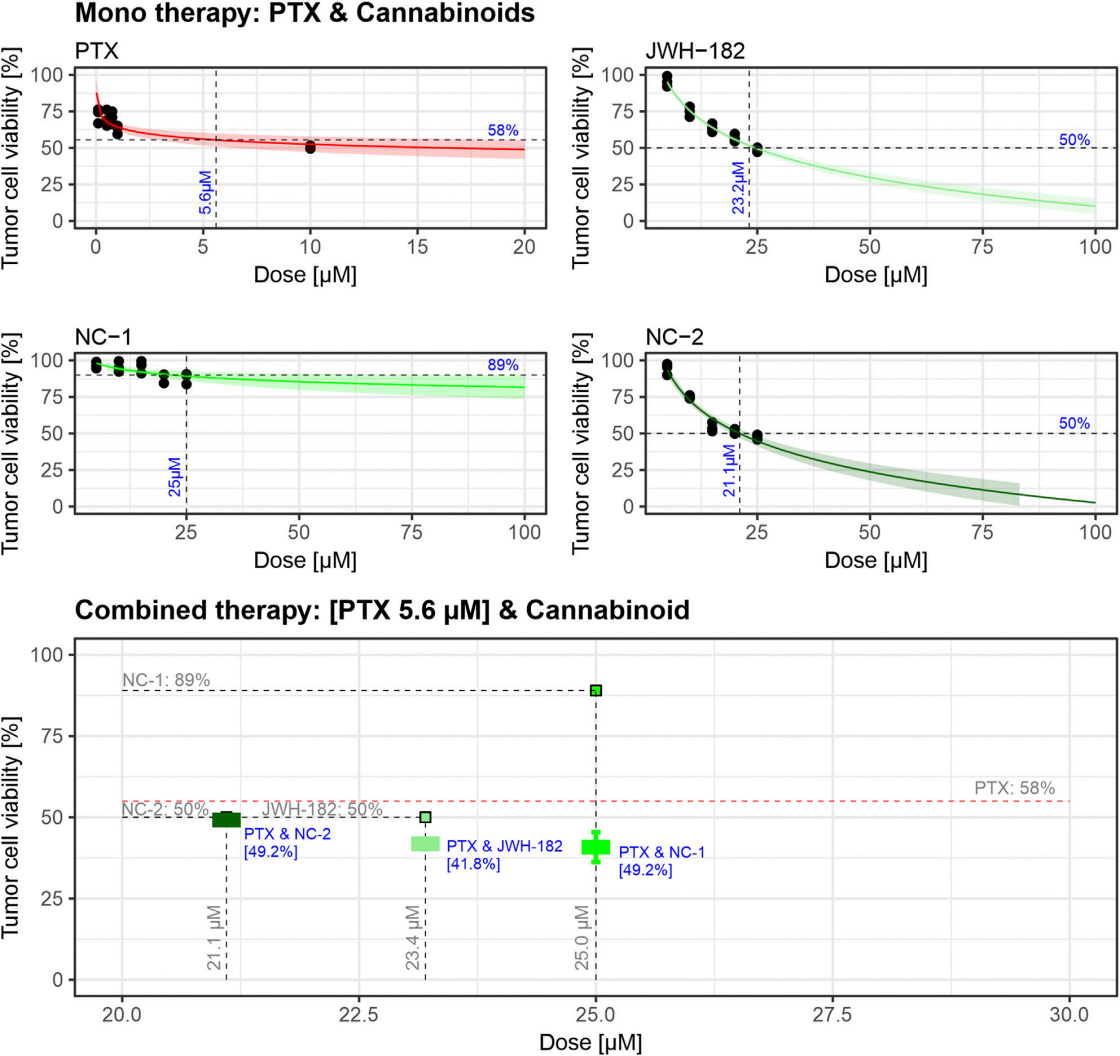
Dose-response curves indicate that PTX exhibits cytotoxic activity with an IC50 of 5.6 µM, while JWH-182 and NC-2 show moderate antitumor effects, with IC50 values of 23.2 µM and 21.1 µM, respectively. NC-1 displays minimal antitumor activity, achieving only 11% cell viability reduction at its highest dose. Combined treatments reveal that cannabinoids enhance PTX’s cytotoxic efficacy, with the PTX & JWH-182 combination showing the greatest synergistic reduction in tumor cell viability. The experiment was performed in triplicate using the 4T1 breast cancer cell line.

4T1 tumor cells were treated for 24 h with increasing concentrations of cannabinoids or paclitaxel (PTX). As shown in Figure 5, cell viability decreased with PTX (IC_50_ = 5.6 µM), as well as with JWH-182 (IC_50_ = 23.2 µM) and NC2 (IC_50_ = 21.1 µM). NC1 showed minimal antitumor effect, with 89% viability at the highest dose. When combined with PTX at IC_50_ doses, all cannabinoids enhanced its cytotoxicity, reducing cell viability below 50%, indicating a synergistic antitumor effect.

#### 3.2.2 *In vivo* assessment

**FIGURE 6:**
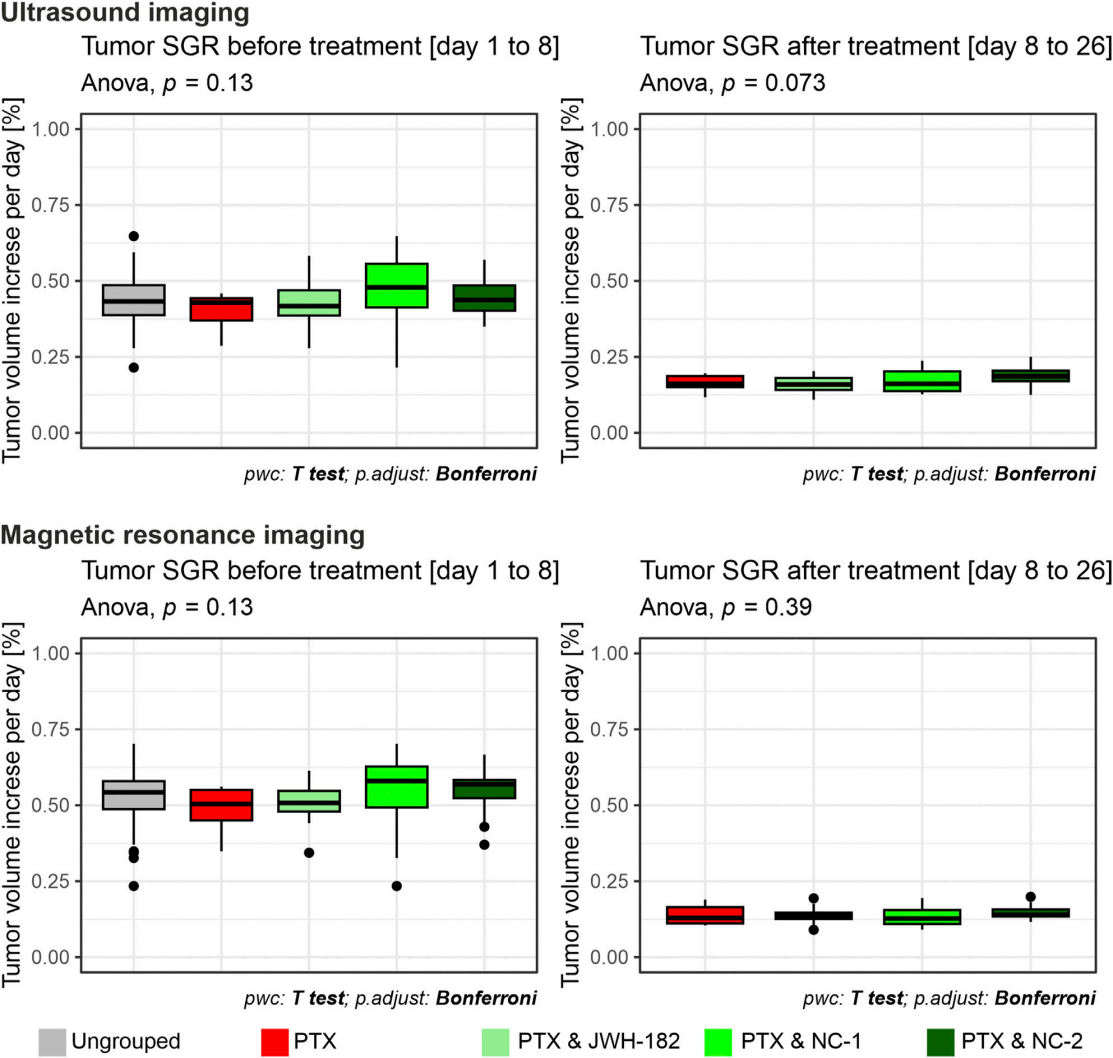
Tumor volumes measured with breast ultrasonography and MRI imaging, results expressed as tumor-specific growth rate (SGR), which represents % of tumor volume increase per day, assessed at baseline (left) and at the end of the treatment period (right). Using both imagining techniques, at baseline (left) all groups showed comparable SGR values, indicating uniform tumor burden prior to treatment initiation. By the end of the treatment period, no significant differences in SGR were observed between the PTX group and the cannabinoid-treated groups (p = 0.073; p = 0.39).

Breast tumors were induced in BALB/c female mice using the mentioned method. The breasts were daily monitored until a firm nodule appeared (day 8), where tumor volumes ranged between 20 and 150 mm^3^. Breast ultrasound was performed to characterize the tumor nodule, which appeared as a hypoechoic nodular lesion, ‘taller than broader’ with spiculated margins, characteristic for tumoral tissue (Gokhale, 2009). Tumor volumes were assessed with breast ultrasound and MRI scans at baseline and after treatment administration, by measuring the antero-posterior, cranio-caudal and transverse diameters. At the end of the experiment, tumor volumes were increased compared to baseline values across all treatment groups. However, for a result which interprets better the pattern of growth of tumors, we chose to calculate the differences in tumor dimensions between baseline and after treatment values, using the tumor-specific growth rate, expressed as the percentage increase in tumor volume per day, as described in the methods section. Prior to treatment (days 1–8), no significant differences in tumor SGR were observed between groups in each imaging technique performed (p = 0.13), indicating comparable baseline tumor growth across all groups. Following treatment (days 8–26), a reduction in tumor SGR was observed across all treatment groups compared to PTX only treated animals, although the differences between the control and cannabinoid groups did not reach statistical significance (p = 0.073, p = 0.39) (Figure 6). Of note, we did not identify using imaging metastasis in either the liver or lungs of animals, regardless of the group.

#### 3.2.3 Histological metastasis rate

At the end of the study, tumors, liver, and lungs were collected and processed for H&E staining. Tumor histology confirmed malignancy, showing dense peripheral zones with large tumor cells, fibroblasts, collagen deposition, fibrosis, and immune infiltration. Transitional zones exhibited pleomorphic, hyperchromatic nuclei, while central areas showed reduced cell density, apoptosis, and necrosis in about one-third of samples.

**FIGURE 7:**
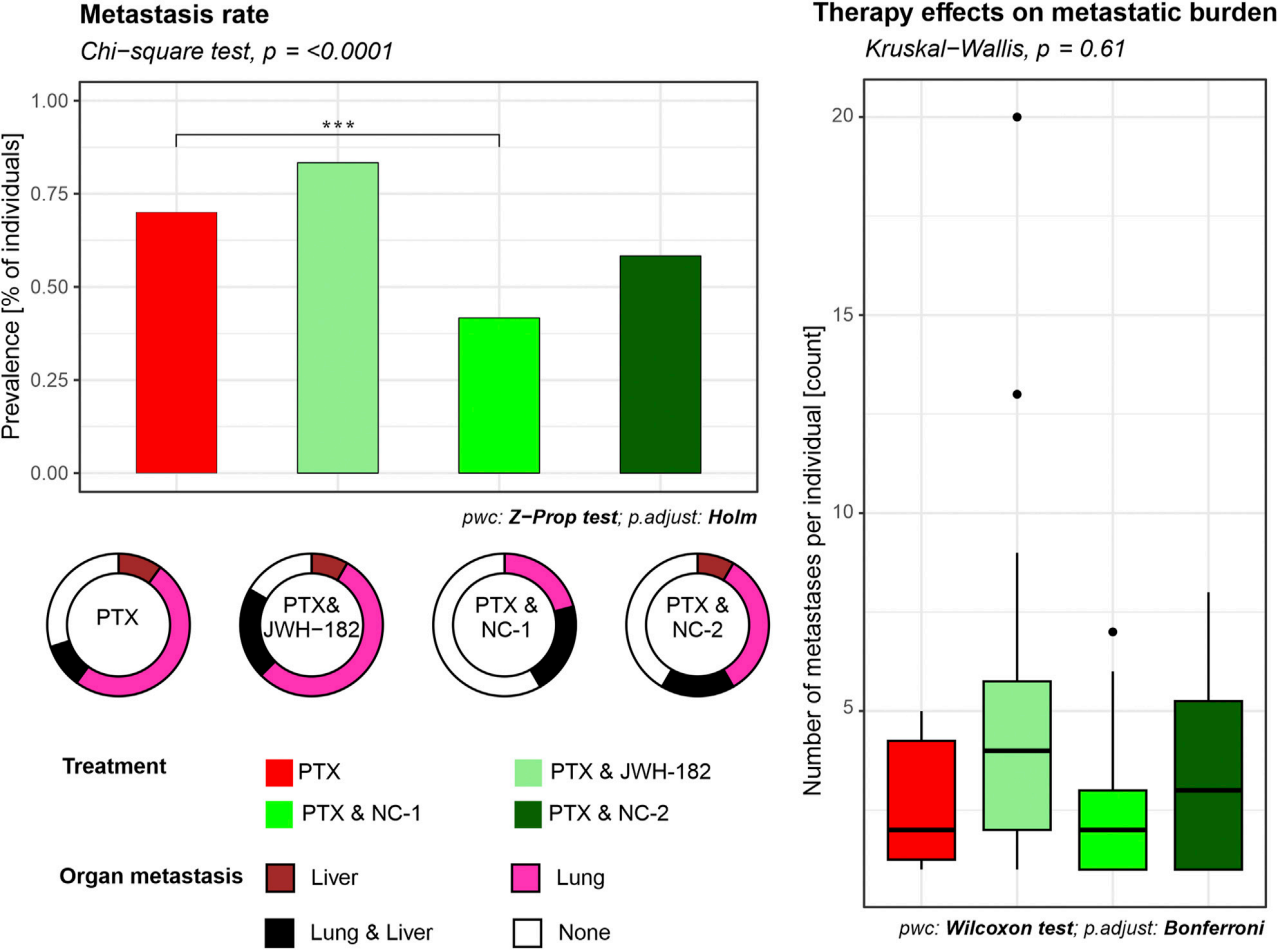
Quantification of metastatic burden in liver and lung tissues. Metastasis rates were determined from histological analysis of H&E-stained sections of organs from each animal. The percentage of animals presenting with at least one metastatic lesion per organ was calculated for each experimental group. Results are either presented as prevalence of metastasis (% of individuals), where NC1 presented with a decrease in prevalence (p < 0.001), or number of metastases per individual.

Although MRI showed no metastases, histological analysis revealed secondary tumor foci in the liver and lungs, confirming hematogenous spread. Among treatment groups, JWH-182 showed the highest metastatic burden (83.3%), even exceeding controls (70%), suggesting a possible protumor effect. NC1 had the strongest antimetastatic impact, lowering metastasis to 41.7%, while NC2 reduced it to 58.3%. Lung metastases were most common in PTX and PTX + JWH-182 groups, whereas PTX + NC1 and PTX + NC2 groups had fewer lesions in both lungs and liver (Figure 7).

### 3.3 Cancer-associated cachexia

**FIGURE 8:**
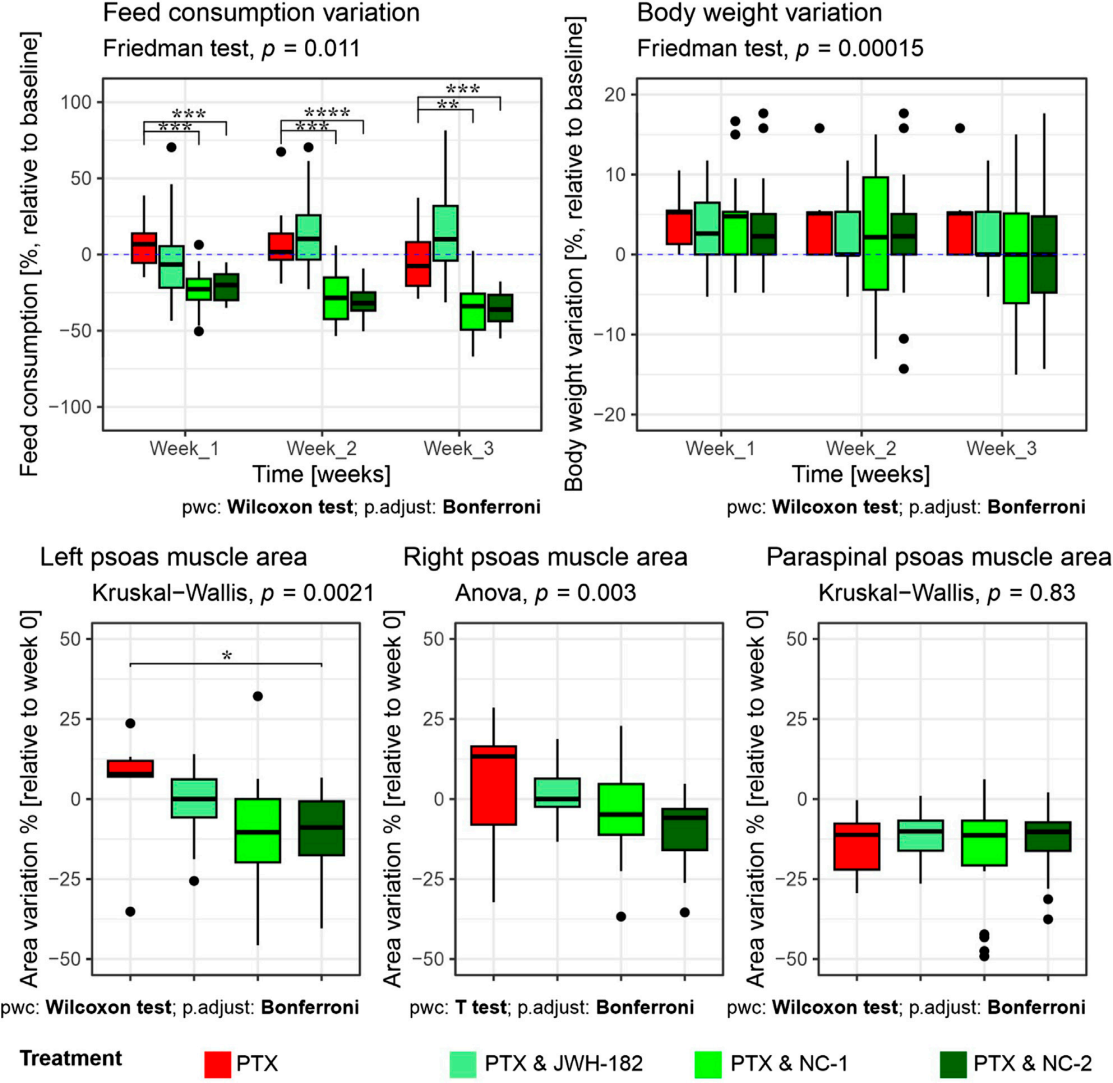
Evaluation of cancer cachexia parameters in treated animals. Top row: Variation in food consumption (left) and body weight (right), expressed as percentage relative to baseline (week 0), across 3 weeks of treatment. Animals receiving PTX alone showed a significant decrease in food intake, which was partially reversed by JWH-182 (p < 0.01 at week 3), whereas NC1 and NC2 exacerbated appetite suppression (p < 0.001). Body weight remained stable across all groups, with no statistically significant intergroup differences throughout the study. Bottom row: MRI-based assessment of skeletal muscle mass, expressed as percentage change from baseline. A significant reduction in left psoas muscle area was observed only in the PTX & NC2 group compared to PTX alone (p < 0.05); no significant differences were detected in the right psoas or paraspinal muscles across treatment groups.

Cancer cachexia was evaluated by analyzing three aspects: appetite, body weight and MRI-assessed sarcopenia of psoas and paraspinal muscles, as presented in Figure 8.

Feed consumption varied significantly across groups and timepoints. Animals treated with PTX alone exhibited a progressive decline in food intake relative to baseline, reaching a significant reduction by week 3. Notably, the co-administration of PTX and JWH-182 was associated with a partial reversal of this anorectic effect, resulting in higher food intake by the third week compared to PTX-only, however not statistically significant. In contrast, both NC1 and NC2 exacerbated the anorexia-like phenotype, showing a more pronounced and statistically significant suppression of feed intake by week 3 (PTX & NC1 vs. PTX, p < 0.001; PTX & NC2 vs. PTX, p < 0.0001), suggesting a potential anorexigenic effect of these cannabinoid analogues in this context.

Despite significant intergroup differences in food consumption, no corresponding variations in body weight were observed among the groups over the 3-week study period (p = 0.00015). All treatment groups, including PTX alone and the three cannabinoid combinations, maintained stable body weights, with only minor fluctuations around baseline.

MRI-based quantification of skeletal muscle areas provided deeper insight into cancer-associated sarcopenia, revealing group-specific muscle loss. Although numerical differences were observed across groups, a statistically significant reduction in muscle area was only detected in the left psoas muscle of animals treated with PTX & NC2 compared to PTX alone (*p* < 0.05). For the right psoas and paraspinal muscles, no significant differences were identified between treatment groups, indicating that the overall impact of cannabinoids on MRI-assessed sarcopenia was limited and muscle loss was not consistently modulated by any treatment.

### 3.4 Toxicity and tolerance

#### 3.4.1 Blood work

**FIGURE 9:**
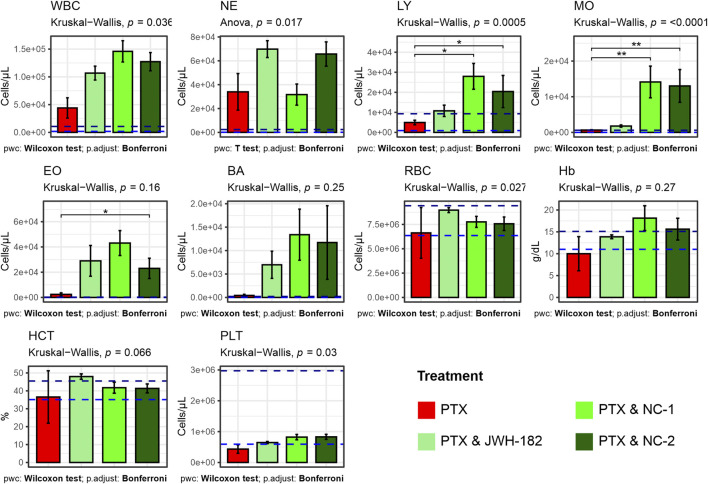
Results of complete blood count (white blood cells, neutrophils, lymphocites, monocytes, eosinophils, basophils, red blood cells, hemoglobyn, hematocrit, platelets) performed at the end of study treatments. Increased white blood cells among all groups caused by leukemoid reaction, produced by the 4T1 cell line. High levels of lymphocites among NC1 and NC2 treated animals.

Blood analysis (Figure 9) showed elevated leukocyte counts in all groups, indicating tumor-related inflammation (Yavari et al., 2023). However, significant differences in lymphocyte levels (p = 0.0005) suggest an immunomodulatory effect of cannabinoids. PTX alone reduced lymphocytes, while NC1 and NC2 increased them, possibly reflecting enhanced immune function. Monocyte and neutrophil counts were also higher in cannabinoid-treated groups (p < 0.0001 and p = 0.017). Platelets, reduced by PTX, were partially restored, especially with NC2 (p = 0.03). Red blood cell counts were higher in cannabinoid groups vs. PTX alone (p = 0.027), while hemoglobin and hematocrit remained unchanged (p > 0.05), indicating stable oxygen transport.

#### 3.4.2 Organo-somatic index

**FIGURE 10:**
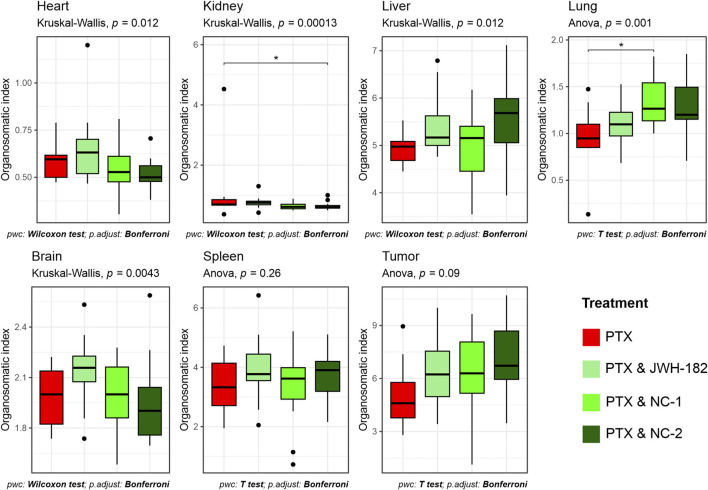
Proportional weight of organs at sacrifice, expressed as organo-somatic index.

At the end of the study, organs were weighed, and organo-somatic indices calculated (Figure 10). NC2-treated animals showed a significant increase in kidney index (p < 0.0001), suggesting renal stress. All treatment groups had elevated spleen indices, consistent with 4T1-induced splenomegaly (Yavari et al., 2023). Tumor weights did not differ significantly across groups (p = 0.09), matching imaging results.

#### 3.4.3 Histopathology report

All groups showed preserved liver architecture with hepatocytes arranged radially around central veins. Isolated clusters of leukemoid cells indicated metastatic status. Three main lesions were observed: congestion, vacuolar degeneration, and inflammation. Congestion was most pronounced in the control and JWH-182 groups, with sinusoidal dilatation and enlarged central veins. NC1 and NC2 groups showed only mild, focal congestion.

Vacuolar degeneration, marked by swollen hepatocytes with clear finely granular that may appear “wispy” or reticulated cytomplasm, and irregular nuclei, appeared in all groups but was more widespread in NC1 and NC2. In control and JWH-182 groups, ballooned hepatocytes were mainly found in centrilobular areas, while in NC1 and NC2 groups, they were more numerous and diffusely distributed across all liver zones.

**FIGURE 11:**
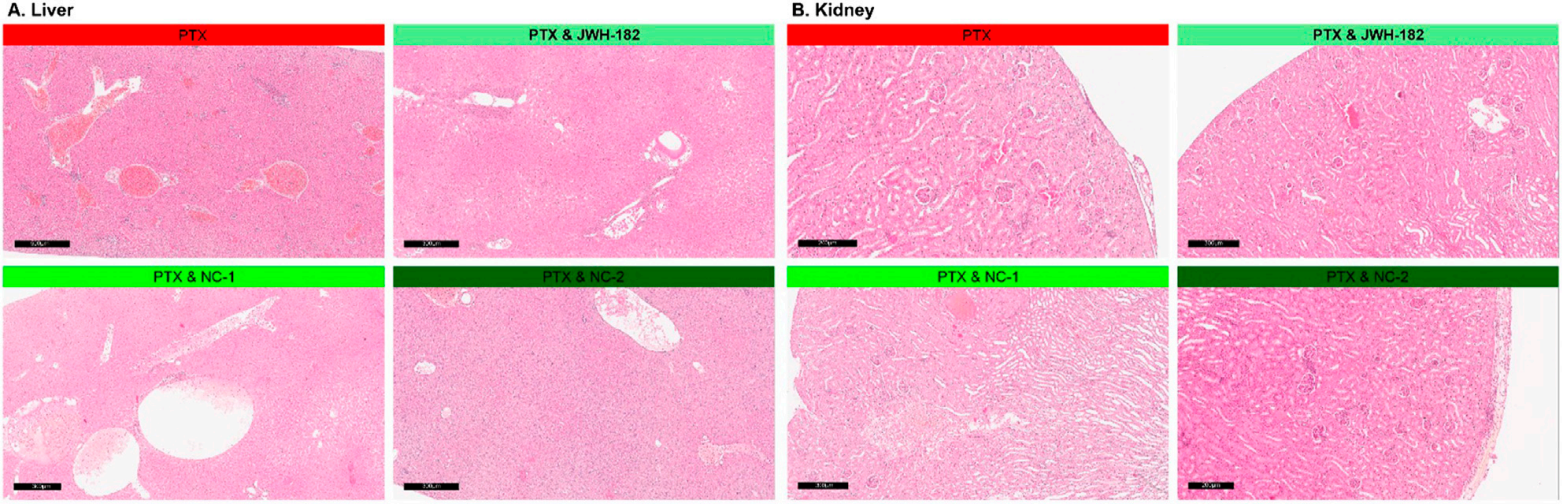
Histopathological aspects of liver **(A)** and kidney **(B)** of animals treated with either JWH-182, NC1 or NC2. H&E staining. Barr200 µm.

Inflammation was intense in control and JWH-182 groups, with mixed infiltrates and microabscesses, especially around central veins. In contrast, NC1 and NC2 showed reduced, focal inflammation. Kidney histology was generally preserved, with cortex and medulla intact. All groups showed glomerular damage, including mesangial proliferation and capillary thickening. NC1 and NC2 also had signs of ischemic injury, shrunken glomeruli with collapsed capillaries and widened Bowman spaces. Tubular damage was mild in controls, while JWH-182 showed loss of brush borders and hyaline casts. NC1 and NC2 exhibited hydropic degeneration, extensive epithelial cell loss, and signs of acute tubular necrosis—more severe in NC2. Vascular congestion was most evident in control and JWH-182 groups. All groups had mild interstitial inflammation and focal tubular atrophy. Details are shown in Figure 11.

#### 3.4.4 Treatment tolerance

**FIGURE 12:**
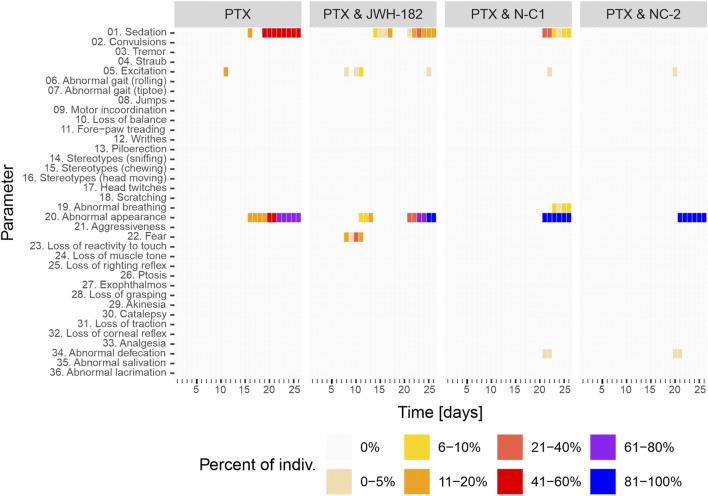
Behavior modifications of study animals throughout the experiment, with results showing a clear animal suffering and low reactivity at the end of study period, in all study groups.

PTX-treated animals showed frequent adverse effects, with sedation in 41%–60% and poor overall appearance in 61%–80% (Figure 12), consistent with known PTX exposure toxicity. JWH-182 reduced these effects, though mild sedation (11%–20%) appeared later in the study. The PTX & NC1 group showed less sedation but some abnormal breathing (∼10%) and worsened appearance, suggesting systemic toxicity. NC2 reversed PTX-induced sedation, but animals still showed deteriorated appearance by the study’s end.

## 4 Discussion

Cannabinoids are widely used by cancer patients, often beyond approved indications. Currently, the only FDA-approved cannabinoids in oncology are Dronabinol® and Nabilone®, used solely for chemotherapy-induced nausea and vomiting (Braun et al., 2024). Despite this, approximately 22% of cancer patients report using cannabis-based products for symptom relief (Amin et al., 2024). The increasing availability of synthetic cannabinoids and new plant strains raises safety concerns, as most lack thorough testing for toxicity and efficacy (Bonn-Miller et al., 2018).

**FIGURE 13:**
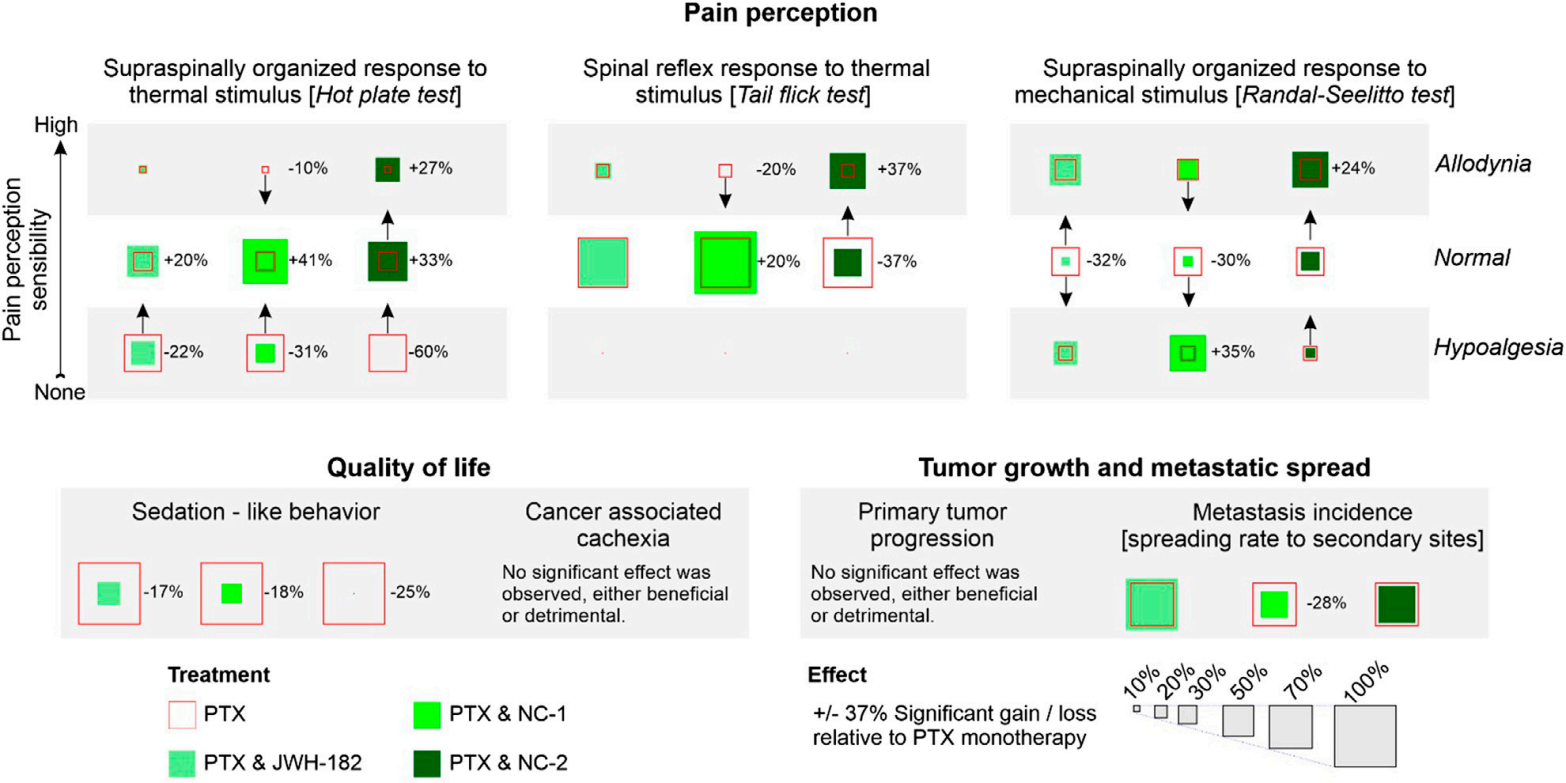
Summary of findings. Added effect.

Our study evaluated one synthetic cannabinoid, JWH-182, and two phytocannabinoid products, Cannabixir® Medium dried flowers (NC1) and Cannabixir® THC full extract (NC2), which had not been previously tested in a cancer context. We found that these cannabinoids provided analgesia in murine models of CIPN, reduced tumor burden, and influenced metastasis. However, these benefits were counterbalanced by decrease tolerance, namely, exacerbated cancer-associated cachexia and liver and kidney toxicity (results summarized in Figure 13).

Firstly, we assessed the analgesic effects of cannabinoids using the Hot Plate, Tail Flick, and Randall-Selitto tests, each targeting different levels of the nociceptive hierarchy. The Hot Plate Test assesses centrally mediated thermal nociception, emphasizing supraspinal processing involving the thalamus, cortex, and descending pathways from the periaqueductal gray and rostral ventromedial medulla (Tjølsen et al., 1991). The significant allodynia and hypoalgesia observed in controls, underscore profound dysfunction in these descending pathways, likely due to PTX-induced neuroinflammation, glial activation, and oxidative stress (Ji et al., 2018). Interestingly, treatment with NC1 and NC2, both THC-dominant formulations, effectively restored normal pain thresholds, likely due to the ability of cannabinoids modulate the neuroinflammatory environment and enhance neuronal survival by protecting the nerves morphology and function, effect previously presented by our group (Filipiuc et al., 2024; Creanga-Murariu et al., 2024). Tail Flick test, evaluates spinally mediated reflexes to thermal stimuli, focusing on spinal nociceptive pathways (Tjølsen et al., 1989). PTX-treated animals exhibited moderate impairments in this test, reflecting central sensitization, a hallmark of CIPN characterized by increased spinal excitability and reduced inhibitory neurotransmission, while NC1 and NC2 suppress spinal hyperexcitability, in line with previous findings (Rahn et al., 2007; Rahn and Hohmann, 2009; Fine and Rosenfeld, 2013). Mechanical nociception was assessed with Randall-Selitto test (Santos-Nogueira et al., 2012), with significant mechanical allodynia and hypoalgesia observed in PTX-treated animals, likely due to axonal degeneration, mitochondrial dysfunction or inflammation (Flatters and Bennett, 2006). NC2 demonstrated superior efficacy in reversing these effects, probably given the ability of THC to reduce peripheral sensitization through cannabinoid receptor modulation in nociceptors or peripheral immune cells, by suppressing the release of inflammatory mediators (Gutierrez et al., 2007). These tests underscore the multi-level modulatory effects of cannabinoids in CIPN, revealing distinct therapeutic profiles. NC1, with strong central activity, appears well-suited for patients experiencing generalized pain and central sensitization. In contrast, NC2’s dual central and peripheral actions may offer greater benefit for those with localized inflammatory pain. Meanwhile, the moderate efficacy of JWH-182 suggests a more limited role, likely confined to spinal nociceptive modulation.

Regarding the antitumor properties of cannabinoids, the *in vitro* test revealed synergic cytotoxic activity with PTX, an already known effect (Velasco et al., 2016a; McAllister et al., 2011). On the other hand, imaging techniques performed *in vivo,* failed to show improvements in primary tumor growth (measured via SGR), aspect supported also by the tumor weight (organo-somatic index), highlighting the complexity of cannabinoid action in a physiological tumor microenvironment (Huber and Mistry, 2024). Despite the lack of direct suppression of primary tumor growth, metastasis rate analysis performed on harvested organs revealed substantial differences among cannabinoid-treated groups. NC1 demonstrated the most pronounced antimetastatic effect, leading to a clear reduction in metastasis incidence compared to both paclitaxel alone and the other cannabinoid treatments, reinforcing the idea that NC1 may actively suppress metastatic dissemination, even though it did not significantly alter the growth of the primary tumor. In contrast, JWH182 was associated with the highest metastasis rate, surpassing that of paclitaxel alone, indicating a potential protumor effect that may facilitate tumor dissemination rather than inhibit it. NC2 showed an intermediate response, with a reduction in metastases compared to paclitaxel alone, though not as effective as NC1.

The contradictory results from imaging and histopathological evaluation suggest that these compounds may influence tumor spread through mechanisms independent of proliferation control. One explanaition is that cannabinoids may influence metastasis through immune modulation, angiogenesis, or extracellular matrix remodeling rather than direct tumor cytotoxicity (Prakash and Shaked, 2024). This is further supported by our hematology assessment results, where we found high levels of circulating lymphocytes among NC1 and NC2 groups, a known target of CB2R agonists (Cabral and Griffin-Thomas, 2009). Platelet counts, significantly reduced in PTX-treated animals, were partially restored in cannabinoid-treated groups, particularly NC2, reflecting an improved hematopoietic function rather than a pro-metastatic shift, as metastasis rates were stable (Liao et al., 2023). RBC counts were also higher in cannabinoid groups, with stable hemoglobin and hematocrit levels, suggesting preserved erythropoiesis possibly due to anti-inflammatory effects mitigating erythropoietin suppression (Baradaran Rahimi and Askari, 2022).

Although we showed clear benefits for CIPN and antitumor activity, contrary to expectations, cannabinoids did not have any benefits for cancer-associated cachexia, a syndrome characterized by systemic inflammation, anorexia, and skeletal muscle wasting (Ni and Zhang, 2020). While PTX reduced appetite (Hariyanto and Kurniawan, 2021), the addition of cannabinoid treatment did not consistently reverse anorexia, nor did it worsened. Interestingly the effects of cannabinoids were different, with JWH-182 slightly improving the food intake by the end of experiment, while NC1 and NC2 showing results more prone to an anorexigenic effect. Although NC1 and NC2 significantly reduced food intake, these differences did not result in measurable body weight loss, indicating the presence of compensatory metabolic adaptations. Such adaptations may involve altered energy partitioning, improved nutrient utilization, or mobilization of alternative substrates that maintain energy balance over short observation periods (Fearon et al., 2011). Similar mechanisms have been described in preclinical cachexia models, where decreased intake can be offset by reduced energy expenditure, enhanced gluconeogenesis, or shifts in lipid and protein metabolism, thereby delaying overt weight (Tisdale, 2009; Argilés et al., 2015). These interpretations are further supported by MRI-based assessments of muscle area, which revealed no consistent or widespread evidence of sarcopenia across treatment groups. The sole exception was observed in the left psoas muscle of animals treated with NC2, where a statistically significant reduction in muscle area was detected. This finding should be contextualized anatomically, as the tumor was inoculated near the fifth left mammary gland, potentially contributing to localized effects on adjacent musculature.

Overall, these findings suggest that cannabinoids may selectively influence certain features of cancer cachexia—most notably, feeding behavior—without uniformly inducing clinically significant weight loss or skeletal muscle atrophy over the short term. This pattern offers a degree of reassurance regarding their safety profile in cachectic settings, particularly when weighed against their demonstrated benefits in neuropathic pain relief and antitumor efficacy.

When speaking about drug-induced toxicity profile of cannabinoids, data derived from the histopathological evaluation of renal and hepatic tissue alterations (Kleiner, 2017; Radi, 2019). In the liver, while the general histoarchitecture remained preserved, three main pathological features were observed: congestion, hepatocyte vacuolar degeneration, and inflammation. Animals treated with JWH-182 exhibited severe sinusoidal congestion, medium hepatocyte ballooning (particularly in centrilobular areas), and a marked inflammatory response with microabscess formation, suggesting the increase of vascular remodelling and decrease of innate and acquired immunity. In contrast, NC1 and NC2 groups showed reduced congestion and inflammation but widespread vacuolar degeneration, randomly affecting hepatocytes across all metabolic zones, indicating the absence of an adaptive capacity to cytotoxicity, with disturbances in ionic and fluid homeostasis. In the kidney, all groups displayed glomerular damage including mesangial proliferation and capillary wall thickening, varying degrees of ischemic injury and tubular damage, with similar extent of vascular congestion and inflammation. JWH-182 induced a limited number of ischemic renal corpuscles and maintained tubular histology associated with some apoptotic features and discrete epithelial damage, indicating a lower renal cytotoxicity compared to NC1 and NC2 groups, where almost all renal corpuscles were atrophic and the tubules presented severe epithelial degeneration, hydropic changes and acute tubular necrosis, causing a greater susceptibility to ischemic and necrotic damage. The variation in lesion patterns suggests distinct mechanisms by which PTX, alone or combined with natural or SCs, impacts detoxification systems. Our findings indicate that cannabinoids modulate chemotherapy-induced toxicity, with JWH-182 amplifying inflammation and vascular stress, while NC1 and NC2 disrupt cellular homeostasis and heighten susceptibility to renal ischemia.

The strengths of this study lies in its comprehensive design, integrating validated neuropathic pain models and test, advanced imaging modalities, such us whole-body MRI and breast ultrasonography, and detailed histopathological assessment to investigate cannabinoid effects in a clinically relevant manner, using a murine model of breast cancer with paclitaxel-induced neuropathy. An important methodological strength is the use of GMP-certified cannabinoid formulations, which ensure standardization, reproducibility, and greater translational validity compared to non-standardized preparations commonly reported in the literature. Nevertheless, the relatively short observation period may not adequately capture long-term effects of cachexia, survival, or delayed toxicities, while the fixed-dose approach does not account for potential dose–response heterogeneity. Furthermore, interspecies pharmacokinetic and metabolic differences limit the direct application of these findings to human oncology. Despite these constraints, the results support the potential role of cannabinoids as adjuvant therapies for chemotherapy-induced neuropathic pain and modulators of tumor dissemination, while emphasizing the necessity of careful clinical monitoring for appetite alterations and organ-specific toxicities, justifying the translation to human studies.

## 5 Conclusion

Cannabinoids show promise as supportive agents in oncology, particularly in alleviating CIPN and modulating tumor dissemination, while their effects on cachexia appear limited but largely neutral. Safety concerns, especially hepatic and renal toxicity, warrant careful attention, and future clinical studies should balance analgesic and antitumor benefits with rigorous safety monitoring. Overall, the use of GMP-certified formulations enhances translational relevance and supports further preclinical and clinical evaluation.

## Data Availability

The original contributions presented in the study are included in the article/supplementary material, further inquiries can be directed to the corresponding author.
